# Synergism of Synthetic Sulfonamides and Natural Antioxidants for the Management of Diabetes Mellitus Associated with Oxidative Stress

**DOI:** 10.3390/cimb47090709

**Published:** 2025-09-01

**Authors:** Ancuța Dinu (Iacob), Luminita-Georgeta Confederat, Ionut Dragostin, Ionela Daniela Morariu, Dana Tutunaru, Oana-Maria Dragostin

**Affiliations:** 1 Research Centre in the Medical-Pharmaceutical Field, Department of Pharmaceutical Science, Faculty of Medicine and Pharmacy, “Dunarea de Jos” University of Galati, 800201 Galati, Romania; ancuta.dinu@ugal.ro (A.D.); ionut.dragostin@ugal.ro (I.D.); dana.tutunaru@ugal.ro (D.T.); 2 Department of Biomedical Sciences, “Grigore T. Popa” University of Medicine and Pharmacy of Iasi, 700115 Iasi, Romania; georgeta-luminita.confederat@umfiasi.ro; 3 Department of Environmental and Food Chemistry, Faculty of Pharmacy, “Grigore T. Popa” University of Medicine and Pharmacy, 700115 Iasi, Romania; ionela.morariu@umfiasi.ro

**Keywords:** type 2 diabetes mellitus, oxidative stress, sulfonamide, antioxidant, synergy

## Abstract

In the context of expanding research on the development of compounds with multiple therapeutic actions, this study aims to consolidate findings from the last decade on new synthetic sulfonamide therapies for managing type 2 diabetes mellitus (T2DM) associated with oxidative stress (OS). The novelty of this synthesis study lies in the synergistic approach of antidiabetic molecular targets with those against oxidative stress, having the sulfonylurea class as a common point. By utilizing international databases, we identified and selected conclusive studies for this review. Promising results have been achieved through dual therapies that combine antioxidants (such as sesame oil, naringin, alpha-lipoic acid, resveratrol, and quercetin) with sulfonylureas (including glipizide, glibenclamide, gliclazide, and glimepiride). Additionally, triple therapies that associated sulfonylureas with other classes of antidiabetic medications have also shown encouraging outcomes. These findings are supported by in vivo tests conducted on experimental laboratory models as well as on human subjects. These recent advancements in synthetic sulfonamide research point to a promising future in diabetes management, especially considering the dual functionalities demonstrated by in vivo studies—specifically, their antidiabetic and antioxidant effects. Moreover, the synergy between sulfonamides and other antioxidant agents represents a beneficial strategy for optimizing future chemical structures, potentially allowing for their integration into personalized treatments aimed at combating T2DM.

## 1. Introduction

**Figure 1:**
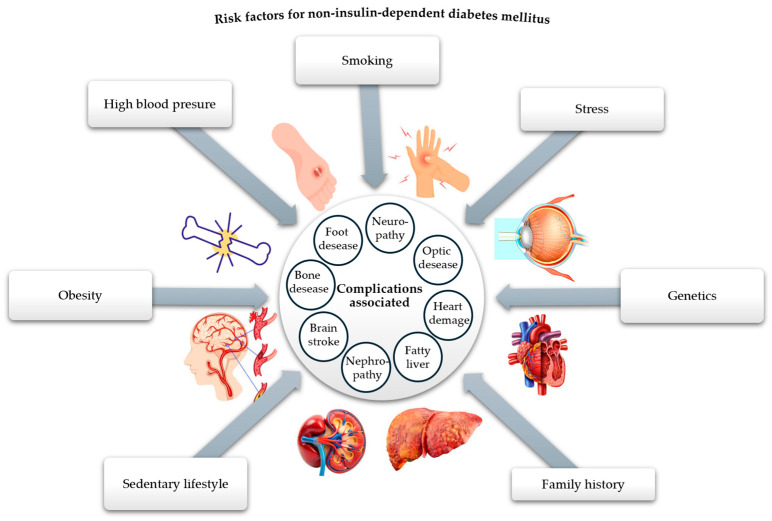
Risk factors responsible for the onset of T2DM and related complications (adapted and modified from [21]).

Undoubtedly, diabetes mellitus (DM) is recognized as one of the most pressing health issues. According to the 10th edition of the IDF Diabetes Atlas, published in 2021, a global prevalence of 10.5% was reported among people aged 20 to 79, with an estimated increase to 12.2% by 2045 [1]. There are many types and forms of diabetes mellitus, the main classification criterion being the cause and mechanisms of production, resulting in type 1 diabetes mellitus (insulin-dependent), type 2 diabetes mellitus—T2DM (non-insulin-dependent), gestational diabetes mellitus, and hybrid forms [2]. Among the forms of diabetes, the non-insulin-dependent type is the most common, affecting around 90% of people with diabetes, which is why we are interested in addressing it. This type of metabolic disease is characterized by tissue resistance to insulin and an imbalance in the pancreatic β-cells, which secrete insufficient insulin [3]. The high prevalence of this type of diabetes in the population has led to a reduced life expectancy, primarily due to the complications it causes at the micro- and macrovascular level (neuropathy, retinopathy, nephropathy, coronary heart disease, heart failure, myocardial infarction, ulcerations, bacterial and fungal infections, kidney disease), as well as due to the triggering factors of this disease (obesity, sedentary lifestyle, oxidative stress, family history, diet) [4,5,6,7,8,9,10,11,12,13,14,15,16,17,18,19,20], as shown in Figure 1.

**Figure 2:**
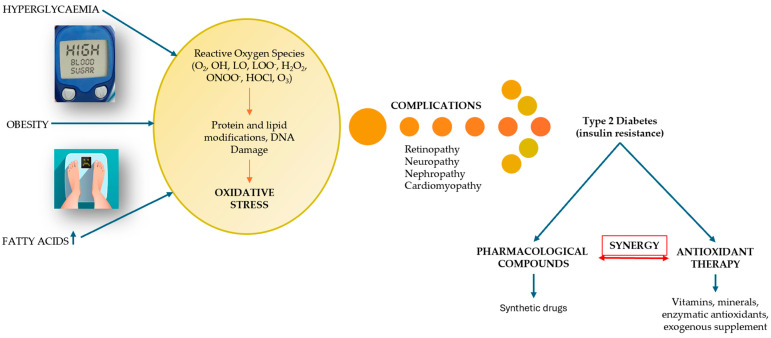
The relational dependence between DM and OS (adapted and modified from [27]).

The peak of recognition of these complications of diabetes was reached during the SARS-CoV-2 pandemic, highlighting that diabetes is one of the leading causes of death worldwide [22]. On the other hand, the risk factors contributing to the onset of DM are passed down from one generation to the next, interacting with each other and being addressed in the literature concerning the incidence and prevalence of DM [5]. One of these factors that has left its mark on the evolution of DM is oxidative stress (OS). The link between OS and DM, illustrated in Figure 2, explains the imbalances that occur between the production of reactive oxygen species (ROS), i.e., free radicals and non-radical molecules, and the body’s reduced antioxidant defense capacity [23]. OS occurs when the production of free radicals and other highly reactive molecules exceeds the body’s ability to counteract them, thus disrupting normal physiological processes and resulting in cellular dysfunction. OS plays an important role in the pathogenesis of DM and is currently an area of interest for researchers [24,25]. Recent research examines the progression of T2DM, highlighting the increased production of ROS due to hyperglycemia and the resulting imbalance between ROS production and the cellular antioxidant system [26].

Concerns about T2DM management date back to the 1950s, when metformin and sulfonylureas formed the cornerstone of DM treatment development [28]. Subsequently, researchers focused on the rapid development of therapeutic targets with increased efficacy through chemical synthesis (starting from already established substances), while others identified opportunities for improvement in the care of people with diabetes, leading to the use of artificial intelligence (AI) applications for early diabetes management, screening, and treatment of comorbidities [29]. Since empathy is a key factor that AI applications cannot offer the patient, the approach to patients with T2DM is still carried out by medical staff, where the frequent recommendation is the administration of oral therapies.

The recognized pharmacological options for treating T2DM are 90% dedicated to oral administration and classified according to their mechanism of action, including the following classes of compounds: sulfonylureas (first and second generation, such as tolbutamide, glibenclamide, gliclazide, glimepiride, glipizide), meglitinides (repaglinide, nateglinide), biguanides (metformin being the most common), thiazolidinediones (TZD) (rosiglitazone, pioglitazone), α-glucosidase inhibitors (acarbose, miglitol, voglibose), dipeptidyl peptidase-IV (DPP-4) inhibitors (sitagliptin, saxagliptin, vildagliptin), dopamine agonists (bromocriptine), sodium-glucose cotransporter-2 (SGLT-2) inhibitors (empagliflozin, dapagliflozin, ertugliflozin), bile acid sequestrants (colesevelam), oral glucagon-like peptide-1 receptor agonists (GLP-1) (like semaglutide) [30].

Currently, many of these drugs are used as monotherapy or in combination with agents from other classes of antidiabetic drugs that have different mechanisms of action or even with agents from different classes, such as those in the antioxidant class (e.g., resveratrol, quercetin, vitamin E, curcumin, N-acetylcysteine, alpha-lipoic acid, etc.), offering a number of beneficial advantages in the management of T2DM [31,32]. The main advantages highlighted by combining antidiabetic compounds with antioxidants refer to: improving glycemic control, counteracting chronic complications of diabetes, protecting pancreatic β-cells, and reducing doses of synthetic antidiabetic drugs [33]. The right synergy between antidiabetic and antioxidant drugs can lead to a more complete pharmacodynamic profile and additive effects in reducing glucose levels and OS [27]. Considering the advantages that the sulfonylurea class has shown over time, namely their widespread use by less developed countries due to their efficiency and low cost, this paper highlights therapeutic combinations based on synthetic antidiabetic compounds with a sulfonamide structure and antioxidant compounds, the latter representing an economically accessible strategy for optimizing treatment [34,35]. Thus, the synergy created could contribute substantially to the development of strategic plans for personalized medicine, with an emphasis on individualizing treatment according to the patient’s metabolic profile. The lack of centralized evidence presenting the optimal doses, the most effective combinations, and long-term safety regarding the synergy of synthetic sulfonylureas with antioxidants are the reasons that led to the development of this paper.

Finally, this review is supported by the analysis of pharmacological and clinical arguments that prove the synergy between synthetic sulfonylureas and antioxidants by evaluating the mechanisms of action and potential benefits, thus offering valuable perspectives for future research that will pave the way for new therapeutic approaches for the management of T2DM associated with OS and related complications.

## 2. Diabetes Mellitus Associated with Oxidative Stress

### 2.1. Pathophysiological Mechanisms of T2DM and Oxidative Stress

**Figure 3:**
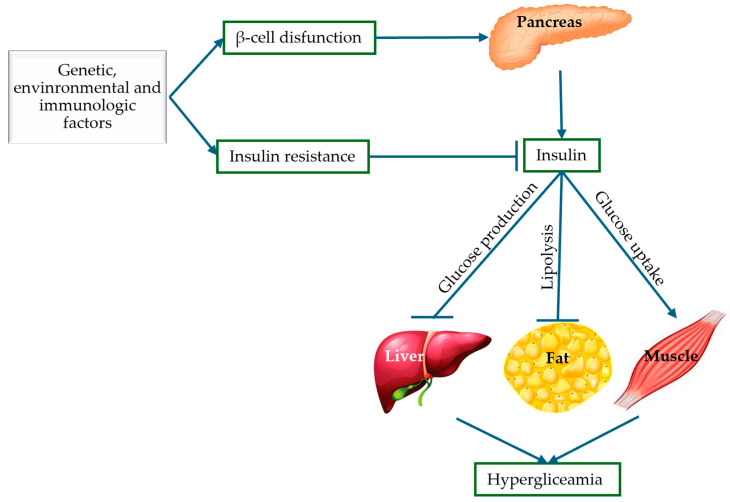
Pathogenic mechanisms of T2DM: insulin resistance and pancreatic β-cell dysfunction (adapted and modified from [37]).

Globally, DM has become a crisis that threatens not only the health of the population, but also the economies of countries. The importance of understanding the pathogenesis of T2DM has proven useful from multiple angles—pharmacological, medical, and research. Thus, the molecular mechanisms of T2DM provide us with information about the impairment of insulin secretion, identifying the following pathological defects: pancreatic β-cell dysfunction and insulin resistance represented by abnormalities in insulin receptors. Knowing the pathogenic mechanisms of T2DM, targeted and rational therapeutic approaches can be identified, such as the use of sulfonylurea drugs when the mechanism of β-cell dysfunction predominates, acting by stimulating insulin secretion [36]. Furthermore, improving insulin sensitivity with antioxidants represents an important therapeutic advantage in the treatment of T2DM. These mechanisms will be discussed in detail in the following subsections and are also illustrated schematically in Figure 3.

#### 2.1.1. Insulin Resistance

This mechanism is defined in a broader sense by the lack of a biological response to normal insulin concentrations. Other authors define insulin resistance in a narrower sense as the reduced level of glucose elimination in sensitive tissues [38]. It has been scientifically proven that insulin resistance is the main cause of the development of syndrome X (a metabolic syndrome), defined as a combination of other conditions: dyslipidemia, hypertension, glucose tolerance, obesity, inflammatory markers, prothrombotic state [39]. The tissues affected by insulin resistance are the liver, adipose tissue, and muscles; and the main consequence of this mechanism is T2DM. However, there are also a number of diseases associated with insulin resistance, such as polycystic ovary syndrome, oxidative stress, and cardiovascular disease [40].

Numerous studies highlight the direct correlation between OS and insulin resistance, making this topic particularly intriguing. The accumulation of ROS in peripheral tissues at the molecular level has become a significant issue. Researchers are actively seeking methods to combat this problem, as oxidation plays a key role in the development of insulin resistance. In their work, Samantha Hurrle et al. emphasize the importance of identifying specific and highly efficient approaches to targeting oxidative stress. They suggest therapeutic combinations that include antioxidants known for their tissue repair properties and their ability to enhance insulin sensitivity, such as tocopherols, ascorbic acid, and alpha-lipoic acid [41].

Insulin resistance is linked to several mechanisms of OS. Key contributors include the mitochondrial production of oxidants like hydrogen peroxide (H_2_O_2_), oxidative phosphorylation, the activation of NADPH oxidase, and the presence of transition metal ions. In their work, Sepiso K. Masenga et al. highlight the most significant mechanisms that lead to insulin resistance, which include the accumulation of lipid mediators such as diacylglycerols and ceramides, mitochondrial dysfunction, inflammation, and the activation of the protein c-Jun N-terminal kinase (JNK). The authors suggest that effective therapeutic strategies to reduce OS and halt the progression of metabolic syndrome should be multidisciplinary. These strategies may include lifestyle changes, pharmacological treatments such as polyphenols found in olives, and surgical interventions [42].

The connection between oxidative stress, insulin resistance, and type 2 diabetes is highlighted by Aikaterini Andreadi et al., who analyzed a new class of hypoglycemic antioxidant enzymes known as peroxiredoxins. This therapy is not only valuable for managing diabetes but also for preventing severe complications, such as cardiovascular diseases, which carry a high risk of morbidity and mortality [43].

There is a research technique that can measure insulin resistance, but it has limited applicability, called the hyperinsulinemic-euglycemic glucose clamp technique [44]. Therefore, insulin resistance can be treated on the one hand by lifestyle changes, with physical activity and diet being important in this case; on the other hand, drug treatment can improve insulin response, thus reducing insulin demand [45].

#### 2.1.2. Pancreatic β-Cell Dysfunction

Another important component in the pathogenesis of T2DM involves abnormalities in the function of β cells in the islets of Langerhans. In this mechanism, insulin undergoes two phases in terms of its secretion in response to glucose administration: an acute phase in which there is a substantial reduction in insulin secretion for several minutes, and a phase in which diabetes is already diagnosed [46].

**Figure 4:**
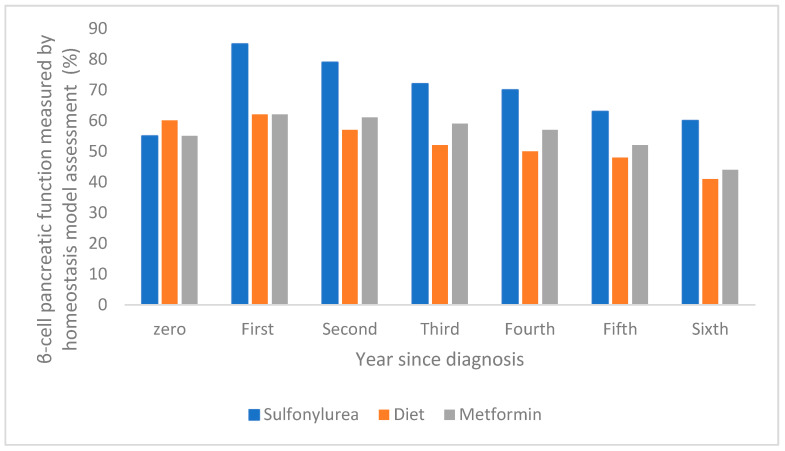
The evolution of pancreatic β-cell function over a one-year period following intensive sulfonylurea therapy compared with metformin and diet (adapted and modified from [47]).

The dysfunction of pancreatic β-cells leads to the onset of two conditions, namely mitochondrial dysfunction and cellular stress in the endoplasmic reticulum, which are influenced by lipotoxicity, glucotoxicity, and chronic inflammation [32]. There are a few factors that accelerate β-cell dysfunction, such as genetic and environmental factors, hyperlipidemia, and hyperglycemia. The literature mentions and exemplifies the exact time when pancreatic β-cell functions are impaired or improved. For example, the evolution of pancreatic β-cell function over a one-year period has been reported in the case of intensive therapy with sulfonylurea antidiabetic drugs, showing a significant increase compared to treatment with metformin or diet, as shown in Figure 4 [47].

OS plays a significant role in the progression of pancreatic β-cell dysfunction, primarily due to low levels of antioxidant enzymes such as catalase and superoxide dismutase. Pancreatic β-cells are particularly susceptible to redox imbalance because they have low levels of antioxidant enzymes. Therefore, OS that leads to β-cell dysfunction involves a complex series of molecular and cellular mechanisms. One key mechanism in this process is the excessive production of ROS (such as superoxide, hydrogen peroxide, and hydroxyl radicals) by mitochondria in β-cells during conditions of hyperglycemia or lipotoxicity [48].

Mitochondrial abnormalities that trigger T2DM occur under conditions of metabolic stress. ROS negatively impact mitochondrial function in several ways: they reduce the efficiency of oxidative phosphorylation, induce apoptosis, and disrupt the production of adenosine triphosphate (ATP). Additionally, impairment of insulin processing and secretion is another mechanism involved in T2DM. ROS disrupt the transport of calcium ions (Ca^2+^), which are essential for insulin exocytosis, affect the phosphorylation of key proteins such as insulin receptor substrate-1 (IRS-1), and also interfere with the function of potassium (K^+^) channels [49].

OS also activates inflammatory pathways, particularly NF-kB (nuclear factor kappa B), which leads to the synthesis and release of pro-inflammatory cytokines such as IL-1β, TNF-α, and IL-6. Additionally, it activates JNK (c-Jun N-terminal kinase) as well as the unfolded protein response (UPR) pathway in the endoplasmic reticulum (ER). The ER is responsible for the proper folding of proteins, including insulin. However, when hyperglycemia occurs, these unfolded proteins accumulate, resulting in the generation of ROS [50,51].

The consequences of these mechanisms reflect apoptosis of pancreatic β-cells, decrease in β-cell mass and function, as well as the establishment and worsening of chronic hyperglycemia.

Research by Park Il Rae et al. highlights a potential solution for addressing pancreatic β-cell damage in type 2 diabetes mellitus (T2DM). Specifically, the glycoprotein CD36 is identified as a key factor that exacerbates oxidative stress; at elevated blood glucose levels, it can induce β-cell apoptosis. This finding points to antioxidants as a promising therapeutic approach [52,53].

A review paper discusses the role of OS and free radicals in diabetes. In Type 1 Diabetes Mellitus (T1DM), OS and free radicals are primarily responsible for the death of β-cells. In T2DM, they contribute to β-cell dysfunction through glucotoxicity and insulin resistance. Additionally, in T2DM, OS affects the development, regeneration, and maturation of β-cells by leading to the loss of essential transcription factors. The authors emphasize the potential of epigenetic therapy and natural antioxidants, such as broccoli sprout extract, sweet chestnut, and curcumin, to alleviate beta cell dysfunction caused by OS [54,55,56,57].

The numerous chronic complications of untreated T2DM have led to and contributed substantially to increased morbidity and mortality worldwide [58,59]. OS, inflammation, mitochondrial dysfunction, diabetic retinopathy, diabetic kidney disease, and cardiovascular disorders are just some of the epigenetic changes that affect tissues, organs, and cells and are caused by high blood glucose levels [60]. The literature also refers to “metabolic memory,” a latent effect that develops complications of diabetes. Authors Hao Dong et al. mention an observational study entitled Epidemiology of Diabetes Interventions and Complications, which is still ongoing and is based on the use of two types of treatment by two groups of diabetic subjects: conventional and intensive early treatment with antidiabetic drugs. It has been shown that diabetics whose blood sugar was regularly controlled with conventional treatment were more prone to complications than diabetics undergoing intensive and early treatment [61].

Thus, two broad categories of complications have been identified: acute and chronic. Acute complications, such as diabetic ketoacidosis, diabetic coma, hypoglycemia, and hyperosmolar hyperketotic coma, can be fatal if not treated properly and identified early [62]. Chronic complications are those that predominantly disrupt the vascular system, both at the micro- and macrovascular levels, affecting the metabolism of proteins, carbohydrates, electrolytes, and fats [2]. Retinopathy, neuropathy, and diabetic nephropathy are chronic microvascular complications, while coronary artery disease, cerebrovascular disease, and peripheral artery disease are chronic macrovascular complications [63]. Extravascular complications such as sexual dysfunction or skin infections may be present.

An active area of research, but one that is not yet fully understood in terms of pathogenesis, progression, and the onset of diabetes complications, is the study of the association between T2DM and one of its key mechanisms, namely OS. Understanding the link between OS and T2DM supports and contributes to the development of effective drug combinations (e.g., sulfonylurea + antioxidants), thus becoming a valuable interdisciplinary topic.

The approach from the perspective of a bidirectional relationship between OS and T2DM is justified both scientifically and clinically. Elevated glucose levels in the early stages of T2DM induce OS, thus OS becomes an important pathogenic link in T2DM that must be combated, otherwise chronic complications, β-cell dysfunction, or the development of insulin resistance will occur [64].

**Figure 5:**
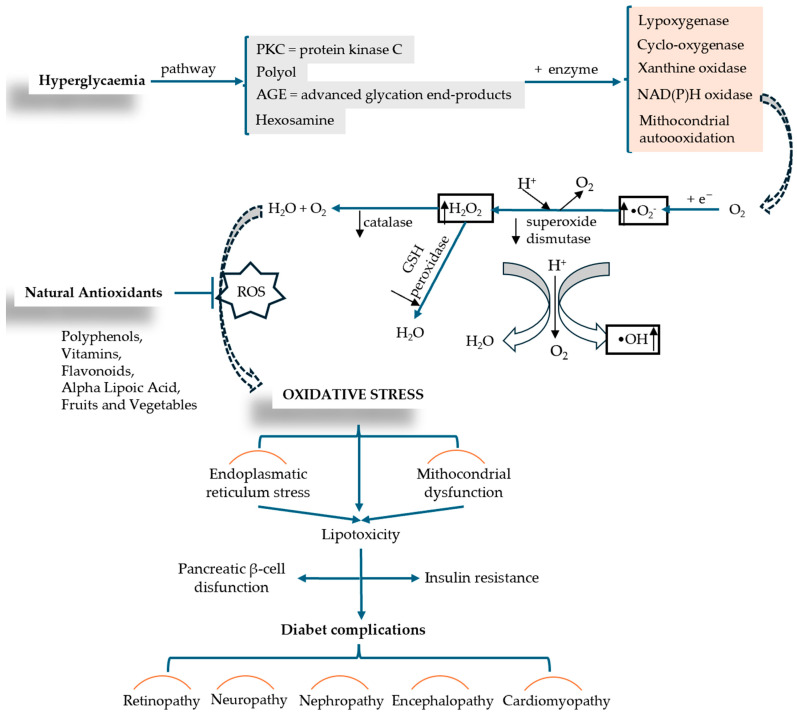
ROS production induced by hyperglycemia and the mechanism of OS installation in relation to diabetic complication (adapted and modified from [66,67]).

The molecular mechanisms involved in OS-associated DM are exacerbated by hyperglycemia through a series of pro-oxidative processes, such as AGEs—the formation of advanced glycation end products; the hexosamine pathway; the polyol pathway; PKC—protein kinase C pathway, glucose oxidation pathway (glycolysis) are shown in Figure 5 [65]. OS occurs when there is an upset between the production of reactive oxygen species (ROS) (oxidative processes) and the body’s ability to neutralize free radicals (antioxidant processes), leading to cellular damage and injury (inflammation, lipid accumulation, fibrosis) [58].

Free radicals, derivatives of O or N molecules, such as hydroxyl (•OH) and peroxide (•O_2_^2−^) radicals, hydrogen peroxide (H_2_O_2_), superoxides (•O_2_^−^), as well as other free radical-generating compounds, are active biomolecules, or ROS, which are physiologically produced by immune cells during metabolic processes [2]. The functions that free radicals perform in molecular processes are related to cell–cell interaction, apoptosis, autophagy, aging, cell proliferation, and memory formation. Also, multiple studies show that OS plays an essential role in metabolic memory in DZ complications, especially in the progression of diabetic rheumatoid disease [3]. The level of free radicals, such as the oxidative damage caused in the body, are measured by OS markers (malondialdehyde—MDA, oxysterols, isoprostanes, nucleic acid oxidation products, reduced glutathione/oxidized glutathione ratio—GSH/GSSG, antioxidant enzymes: superoxide dismutase—SOD, catalase—CAT) [68], These markers which highlight the interdependence between OS and T2DM, represent a pathway for the development and initiation of adjuvant therapies with antioxidant properties, effectively contributing to metabolic control and delaying the onset of complications.

Nadeem Rais et al. conducted a comprehensive review analysis in which they decoded the complex connection between OS and DM, highlighting the mechanisms of interaction between the two conditions and new therapeutic approaches developed to treat the complications that arise. The authors describe a series of processes such as mitochondrial dysfunction and NADPH (nicotinamide adenine dinucleotide phosphate oxidase activity) that lead to excess ROS production and cell destruction. A suggestive diagram (Figure 5) analyzes the mechanism of OS production in relation to diabetic complications. Insulin resistance and pancreatic β-cell dysfunction are caused by factors such as mitochondrial dysfunction and lipotoxicity induced by endoplasmic reticulum stress. As therapeutic interventions, the authors include and describe supplementation with antioxidants (vitamins C and E, beta-carotene, selenium, coenzyme Q10, polyphenols, flavonoids), lifestyle changes, and/or pharmaceutical medications that act as direct antioxidants or enhance the body’s natural antioxidant defense mechanism (N-acetylcysteine, alpha-lipoic acid, metal chelators) [67].

Another recent review focusing on understanding the molecular processes linking SO to DM and the mechanisms by which OS contributes to the onset of DM complications belongs to Xingyu Chen et al. The authors discuss aspects related to hyperglycemic memory and the induction of systemic inflammation, in addition to the mechanisms commonly addressed, namely insulin resistance and insulin production. The authors have identified limitations of small molecules that possess limited antioxidant effects due to their negligible capacity to capture various types of free radicals. This highlights the need for further investigations to discover new and effective therapies [69].

An important antioxidant for neutralizing ROS is glutathione (GSH), the level of which in T2DM is very low, thus leading to inflammation, exacerbation of OS, impairment of redox balance, mitochondrial function, and complications such as neuropathy, nephropathy, and retinopathy. Ioan Dawi et al. present in their paper the combined effects of the antioxidant GSH with vitamin D3 as an anti-inflammatory agent in the management of T2DM, reducing inflammation and attenuating OS, emphasizing the importance of dual-action combination therapy [70].

Alaa AM Osman et al. set out in their work to identify the antioxidant effects of antidiabetic drugs, which they subsequently achieved by collecting information on three classes of antidiabetic compounds widely used in people with T2DM (SGLT2: dapagliflozin, canagliflozin, ertugliflozin, and empagliflozin; GLP-1: semaglutide, liraglutide, dulaglutide; Biguanides: metformin). The author’s review provides an overview of T2DM management and cardiovascular complications through the use of combined antidiabetic pharmaceutical targets with dual effects: hypoglycemic and antioxidant. The antioxidant effects of antidiabetic therapies were centralized from in vivo studies on animal models with induced diabetes and from randomized clinical studies on the human population, mentioning changes in OS biomarkers [71].

### 2.2. Sulfonamide Derivatives: Compounds with Antidiabetic and Antioxidant Potency

Sulfonylureas (SU), remains an area of interest and is still being researched due to its low cost in less developed countries, thus warranting analysis in this paper with the aim of providing an approach that highlights their antioxidant effects both individually and in combination therapies [72,73]. While effective and cost-efficient, this class of drugs has several limitations, particularly when compared to modern treatments. A common issue is the heightened risk of hypoglycemia, which frequently leads to hospitalizations among patients treated with SU [74]. By stimulating insulin secretion independently of blood glucose levels, SU have the disadvantage of promoting excessive glucose storage, which can lead to weight gain—this poses a significant risk for obese patients [75]. In comparison to other modern drug classes, such as GLP-1 receptor agonists and SGLT2 inhibitors, older SU agents lack beneficial cardiovascular effects, meaning they do not reduce the risk of hypertension or myocardial infarction [76]. Furthermore, SU may become less effective after being administered for 1 to 3 years, often necessitating the addition of other antidiabetic medications or even a switch to insulin; therefore, a notable side effect is the decrease in effectiveness over time [77]. Many treatment guidelines emphasize the primary use of SGLT2 inhibitors, GLP-1 receptor agonists, and DPP-4 inhibitors, resulting in a declining use of SU, which are now used less frequently or sometimes replaced by these newer agents [78]. However, the role of sulfonylureas is still well defined in the modern management of Type 2 Diabetes Mellitus (T2DM). SU represent a second-line treatment after metformin for patients with high cardiovascular risk, obesity, and a history of hypoglycemia. They are indicated for young patients with early-onset diabetes when pancreatic β-cells are still functioning and are often combined with metformin for a rapid reduction of blood glucose levels and HbA1c [79].

On the other hand, implementing combination therapies that reassess the SU class presents several practical challenges, including issues related to safety, cost, and education. For instance, while modern therapies like GLP-1, SGLT2, and DPP-4 are expensive, SU are affordable and widely available. This affordability makes them appealing not only in underdeveloped countries but also for patients who lack comprehensive medical insurance [72]. The complexity of combination therapies necessitates careful management to strike a balance between effectiveness and risk. For instance, taking multiple medications as part of a varied daily regimen can lead to decreased adherence to treatment, particularly in patients who are on multiple medications [80]. In these situations, simplified treatment regimens, such as fixed combinations of sulfonylureas, are optimal. Combining SU with metformin or DPP-4 inhibitors helps achieve glycemic targets when monotherapy is no longer sufficient [81]. With over 70 years of use, the effectiveness profile of SU is well-established and predictable, making them easier to manage in practice compared to newer therapies.

Sulfonamide derivatives have a particular affinity for pancreatic β-cell receptors. SU therapy is more reliable and has fewer side effects, with a notable beneficial impact on patients with metabolic diseases prior to 1942, demonstrating low toxicity [82]. It has also been demonstrated that SU have the ability to reduce glycated hemoglobin A1c (HgA1c) to 1.25%, with comparable efficacy [83]. On the other hand, another advantage of this class is its compatibility, thus being used in combination therapies with other antidiabetics or other compounds from other classes [84].

**Figure 6:**
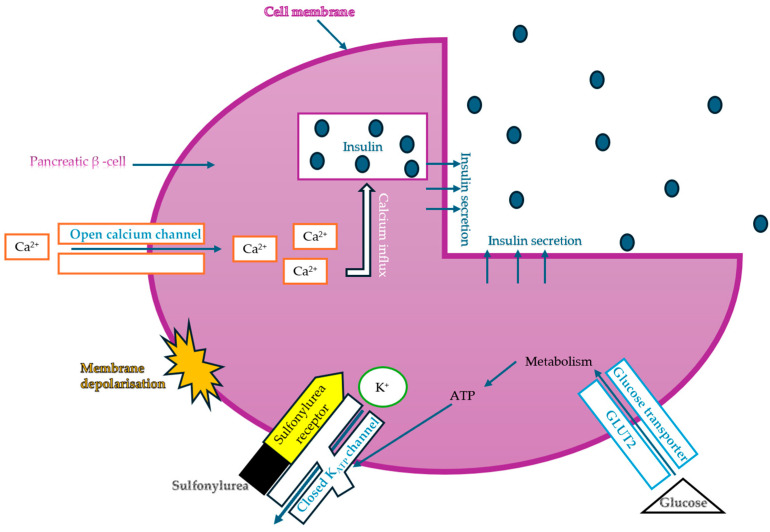
The mechanism of action of sulfonylureas (adapted and modified from [85]).

Considering insulin secretagogues, all generations of SU have the same mechanism of action, namely insulin secretion as a result of stimulation of pancreatic β-cells. The path to understanding the mechanism of action begins with the binding of SU to transmembrane receptors that mediate the blocking of K^+^ (potassium) channels and the influx of Ca^2+^ (calcium) into cells, as illustrated in Figure 6. Therefore, when K^+^ decreases, depolarization of the pancreatic β-cell membrane occurs, thus promoting the opening of Ca^2+^, channels, ultimately stimulating insulin exocytosis. Depending on the types of receptors present in K_ATP_ channels, the affinity of SU compounds varies. SU are eliminated renally, with a high degree of tolerability reported in adult patients. Over time, sulfonamide derivatives have been grouped into three generations based on their evolution, with new derivatives with promising antidiabetic activity subsequently being developed synthetically, as shown in Table 1 [32].

The common feature of sulfonamides is the presence of their functional SU group, which allows binding to the transmembrane receptor (SUR), regulating ATP-sensitive K^+^ channels and thus conferring characteristic hypoglycemic properties.

Scientific research has contributed to important steps taken to develop other compounds in the same class, but with different properties due to altered side chains, thus giving them several improvements in terms of efficacy, safety, tolerance, and potency. Farid M. Sroor et al. developed new sulfonamide derivatives, which are analogs of gliclazide, to evaluate their antioxidant and antidiabetic properties. The starting material for synthesizing these derivatives was p-Tolylsulfonylisocyanate, which reacted with a series of mono-, di-, and tri-substituted anilines. For in vivo testing in laboratory models, gliclazide was used as a reference drug. Favorable results in improving glucose levels were observed for compounds 1c (N-(2,6-dichlorophenylcarbamoyl)-4-methylbenzenesulfonamide) and 5 (N-[(4-methylphenyl)sulfonyl]pyrrolidine-1-carboxamide). The biological properties of these active compounds were enhanced due to the use of silicate-coated nanoparticles, showing improved results compared to gliclazide [86].

A recent study details the procedure for synthesizing certain tolbutamide analogs, specifically 1,2,3-triazole derivatives. After evaluating their antioxidant and antibacterial activities, two compounds demonstrated significant efficacy compared to the standard drug. These compounds are: N-({[1-((2-fluoroethyl)sulfonyl)-1H-1,2,3-triazol-4-yl]methyl}carbamoyl)-4-methylbenzenesulfonamide and N-({[1-((4-chlorophenyl)sulfonyl)-1H-1,2,3-triazol-4-yl]methyl}carbamoyl)-4-methylbenzenesulfonamide. Additionally, the study reported results from in silico testing and molecular docking studies [87].

Therefore, SU are used to treat T2DM, as insulin production is not altered, but rather, insulin resistance is compromised. The antidiabetic activity of sulfonamide derivatives has been evaluated through in vivo studies on animal models with pharmacologically induced diabetes or metabolically induced diabetes (oral glucose tolerance test—OGTT, insulin tolerance test—ITT, basal and postprandial blood glucose, histopathology, etc.), in vivo studies on human subjects (fasting blood glucose, glycated hemoglobin, OGTT, ITT, lipid profile, etc.), in vitro studies (the most common being α-glucosidase and α-amylase enzyme inhibition tests, glucose uptake), in silico studies (molecular docking, quantitative structure-activity relationship, pharmacokinetic prediction—ADME and toxicological prediction—Tox) [88,89,90,91].

For example, starting with 4-aminobenzenesulfonamide, Tanveer Ahmad et al. developed 14 synthetic compounds through reactions with aryl sulfonyl chlorides. The antidiabetic activity of these sulfonylurea-sulfonamide hybrids was tested in vivo using albino mice. The Oral Glucose Tolerance Test (OGTT) was applied, with the synthetic compounds administered 30 min prior to glucose administration. The results were compared to those of glibenclamide, revealing that only six of the synthetic agents (4-Methoxy-N-(4-sulfamoylphenyl)benzenesulfona-mide; 4-[(Propylsulfonyl) amino] benzenesulfonamide; 3-Nitro-N-(4-sulfamoylphenyl) benzenesulfonamide; N-(4-{[(3-Chlorophenyl) carbamoyl] sulfamoyl}phenyl)-4-nitrobenzenesulfonamide; N-(4-{[(3-Chlorophenyl) carbamoyl] sulfamoyl}phenyl)-4-methylbenzenesulfonamide; N-[(3-Chlorophenyl)carbamoyl] -4-[(propylsulfon-yl) amino] benzene sulfonamide) demonstrated a significant reduction in blood glucose levels. This highlights the potential for integrating these compounds into antidiabetic treatments [92].

New synthetic compounds with a sulfonamide structure, derived from 4-aminobenzenesulfonamide, were investigated for their antidiabetic activity through in vitro tests that measured the inhibition of the enzymes α-glucosidase and α-amylase. In the first step, amide groups were synthesized via nucleophilic addition, followed by the incorporation of sulfonylurea groups. The antioxidant activity of these compounds was evaluated using the ABTS, CUPRAC, and DPPH tests; however, the results were not significant. The authors also conducted in silico tests on the compounds [93].

Some representatives of the SU class are recognized not only for their ability to reduce blood glucose levels, but also for their antioxidant capacity, which inhibits the formation of ROS. Thus, relevant studies in the literature emphasize the importance of antioxidant therapy in the prevention or progression of diabetic complications at the micro- or macrovascular level. The scientific literature mentions a notable antioxidant activity for some sulfonamide compounds, as shown in Table 1, evaluated either through in vitro studies (such as DPPH—2,2-diphenyl-1-picrylhydrazyl, ABTS—2,2′-azinobis(3-ethylbenzothiazoline-6-sulfonic acid, FRAP—ferric reducing antioxidant power, ORAC—oxygen radical absorbance capacity, •OH scavenging, cupric reducing antioxidant capacity, lipid peroxidation inhibition test, TPAC—total plasma antioxidant capacity), or through in vivo studies on living biological models (human subjects or laboratory animals) determining the level of OS biomarkers (MDA), evaluating endogenous antioxidant enzymes (SOD—superoxide dismutase, CAT—catalase, GPx—glutathione peroxidase) or glutathione levels (GSH and GSSG), as well as through in silico tests [94,95,96].

**Table 1:** Sulfonamide derivatives grouped according to the order of discovery and their illustration in studies evaluating individually their antidiabetic activity and antioxidant activity (adapted from [97]).

SU (General Chemical Structure)	Generations of Sulfonamide Derivatives	Radical		Studies on Antidiabetic Activity	Studies on Antioxidant Activity
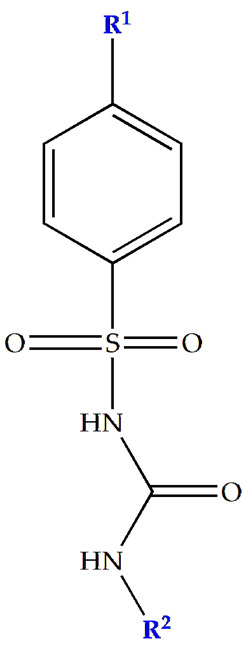		R^1^	R^2^		
	First Generation				
	Tolbutamide	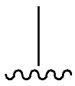	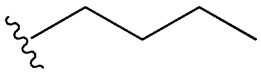	[98]	[87,99]
	Tolazamide	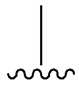	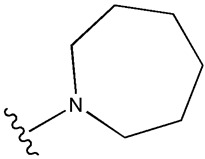	[100]	Not found
	Chlorpropamide	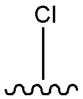	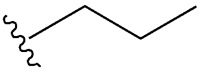	[101]	[102]
	Acetohexamide	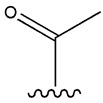	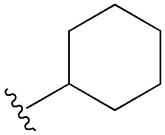	[103]	Not found
	Second Generation				
	Gliburide (sau glibenclamide)	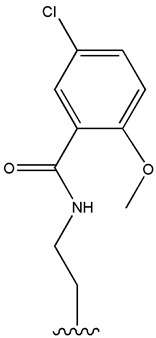	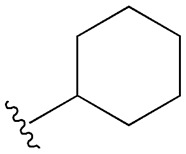	[104,105]	[106,107]
	Glipizide	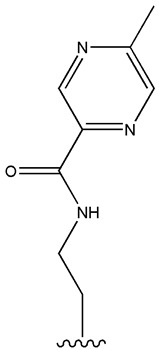	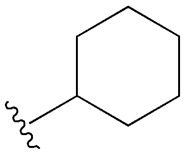	[108]	[109,110]
	Gliclazide	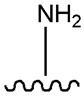	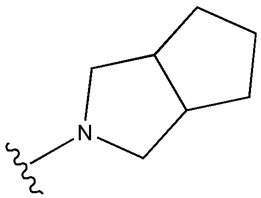	[111]	[112,113,114]
	Third Generation				
	Glimepiride	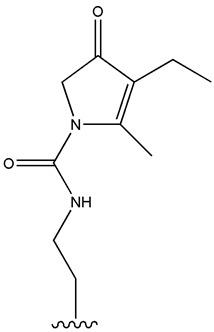	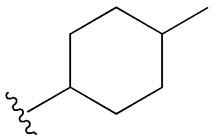	[115]	[116]

Prakash P. Malam et al. included in their study an in vivo evaluation of the effect of glipizide therapy on OS parameters in T2DM. Glipizide testing was performed on 30 diabetic patients for 3 months, with the control group consisting of 30 non-diabetic volunteers. Plasma levels of MDA, SOD, and CAT were evaluated, and the results obtained (MDA and SOD high, and CAT reduced) demonstrated an increase in the antioxidant process, thus reducing OS and implicitly preventing the progression of T2DM and its associated complications [110].

Another study feasible for in vitro testing of the antioxidant activity of antihyperglycemic drugs with a SU structure belongs to Richard O’Brien and his collaborators. In their paper, the authors compare the results obtained by TPAC and low-density lipoprotein (LDL) oxidation tests for gliclazide with those for other SU: glibenclamide, glipizide, tolbutamide, and glimepiride. Also, in the same study, through in vivo tests with gliclazide administration for 10 months by 44 patients with T2DM, it was demonstrated that at a concentration of 1 μM gliclazide, there was a significant inhibition of 8-isoprostanes, specific markers of lipid oxidation, and an increase in SOD, TPAC parameters, thus having notable antioxidant properties with clinical effects [113].

Through in silico molecular docking studies, the free radical scavenging activity of glipizide was evaluated by Safna Hussan K.P. et al. These studies elucidated the binding mode of glipizide through non-covalent interactions, thus opening up a new perspective for the design of powerful antioxidant compounds [109].

### 2.3. Pharmacological Synergies Between Sulfonamide Compounds and Other Pharmacological Agents for the Treatment of DZT2 Associated with OS

Combination therapies have been classified into two categories: dual therapies and multidrug therapies. Multidrug therapies represent a new, broad treatment strategy that involves the use of multiple compounds. This approach poses both challenges and research opportunities for addressing the pathophysiological processes of diabetes mellitus (DM) associated with oxidative stress (OS) [117]. Monotherapies have shown low efficacy, particularly when diabetes is associated with other conditions such as oxidative stress, high blood pressure, and dyslipidemia. Therefore, combination therapies represent a promising alternative for improving lipid profiles, achieving better glycemic control, and delaying disease progression [118].

Through countless studies reported in the literature over several centuries, SU have occupied an important place in the pharmacological arsenal for treating diabetes. The disadvantages of using SU in monotherapy, such as weight gain, gastrointestinal disorders, and the onset of severe hypoglycemia, have been gradually eliminated by combination therapies, with current guidelines promoting the safe use and administration of SU in combination with other drugs, such as antioxidants, other antidiabetics, lipid-lowering drugs, etc. [119]. In this paper, considering treatment options for T2DM associated with OS, combined SU therapies will be centralized and detailed.

In addition to individual testing for the antioxidant activity of SU, new studies present opportunities for combination therapies, where synergistic interactions highlight the need to reduce the dose of antidiabetic drugs and eliminate adverse effects [119,120]. For example, Table 2 summarizes a series of recent therapeutic approaches in the SU class in combination with antioxidants or other classes of drugs for which in vivo tests have been performed, with an emphasis on evaluating synergistic effects and molecular mechanisms of OS attenuation. It is important to mention that there are no in vitro and in silico studies on these combined therapies, but only on monotherapies.

Antioxidant therapy is important for inhibiting or reducing OS caused by excessive ROS production. A healthy body has an antioxidant self-defense system against OS, involving enzymes such as CAT, SOD, GPx, but also non-enzymatic antioxidants such as vitamin C, vitamin E, GSH, bilirubin, and uric acid. The body suffering from metabolic syndrome needs help with antioxidant therapies administered consistently [27]. Of the recognized classes of antioxidants, those most used in therapies associated with SU are polyphenols (curcumin, resveratrol), flavonoids (naringin, quercetin, rutin), vitamin E, alpha lipoic acid, fruits and vegetables (garlic, currants, blueberries, sesame oil, olive oil) [32,121].

For instance, gliclazide—a second-generation oral antidiabetic—is often the preferred choice in situations where weight loss is not a goal or when other oral antidiabetic medications have adverse effects, as it helps mitigate the risk of cardiovascular diseases [122].

The authors Noha F. Abdelkader et al., use a powerful antioxidant, quercetin, in combination with an SU, gliclazide, in their study to evaluate the effectiveness of this therapy on pancreatic β-cells, monitoring changes in lipid levels, glycemic control, oxidative status, and inflammation through in vivo tests on laboratory models, Wistar mice. The medication was administered to diabetic rats for 3 consecutive weeks in batches, as follows: one batch received gliclazide at a concentration of 10 mg/kg, another batch received only quercetin at a concentration of 50 mg/kg, and another batch received a combination of gliclazide/quercetin. The authors found that in the case of combination therapy, there was a favorable synergy between the two substances compared to monotherapy, increasing SOD and reducing GSH, normalizing blood sugar, triglycerides, total cholesterol, and malondialdehyde. These results offer diabetic patients a promising combination for the treatment of T2DM [123].

After analyzing various studies, it was found that combining a sulfonylurea with a proven antioxidant profile, such as gliclazide, and another drug that has antioxidant effects—whether that be another antidiabetic or a natural antioxidant—can be beneficial. This combination therapy helps reduce oxidative stress, prevents complications, improves patients’ quality of life, and contributes to holistic patient management [124].

From a molecular mechanism standpoint, combination therapies that involve sulfonylureas and other classes of compounds provide protection at the vascular, cellular, and mitochondrial levels. These therapies inhibit pro-oxidative pathways—such as the polyol pathway, advanced glycation end-product (AGE) pathway, and protein kinase C (PKC) activation—thereby reducing the production of reactive oxygen species (ROS). Additionally, they enhance the body’s endogenous antioxidant capacity and improve glycemic stability. For instance, gliclazide, which contains a heterocyclic nitrogen nucleus in its structure, inhibits the activation of NADPH oxidase. It also decreases lipid peroxidation and reduces the expression of endothelial adhesion molecules. In combination therapies, gliclazide enhances endothelial function and helps prevent both microvascular and macrovascular complications [125].

On the other hand, there are other antidiabetic drugs with remarkable antioxidant activity, such as metformin. Abdel-Moneim, A.M.H. et al. combined glipizide with metformin to assess oxidative stress, blood glucose levels, and renal function in rats with streptozotocin-induced diabetes. The rats were divided into five groups representing untreated groups, monotherapy groups, and dual therapy groups (glipizide/metformin). The parameters measured were TPAC (total antioxidant capacity), NO (nitric oxide), lipid peroxidation (LPO), blood glucose, glycated hemoglobin, LDL, and HDL. The results suggested several therapeutic benefits of the gliclazide/metformin combination necessary for improving quality of life [83].

**Table 2:** Combined SU therapies for the treatment of T2DM associated with OS.

Therapeutic Combination		Synergistic Effects	Test Performed	General Results	References
SU	Antioxidant/ Antidiabetic				
SU and antioxidants					
Gliclazide 10 mg/kg	Quercetin 50 mg/kg	Immunohistochemical results: restored β-cells number and insulin immunoreactivity to normal values. Morphometric results: normalized number (synergistic effect, *p* < 0.05), area (additive effect, *p* < 0.05) and perimeter (additive effect, *p* < 0.05) in pancreatic islets and beta cells (synergistic effect, *p* < 0.05)	In vivo study diabetic rats	augmented serum superoxide dismutase and reduced glutathione more than gliclazide alone	[123]
Gliclazide 15 mg/kg	Alpha lipoic acid (ALA) 60 mg/kg	Effects on body weight, blood glucose level and pancreas structure: normoglycemia was maintained in comparison with healthy control animals (*p* < 0.05) Effects on inflammation, cardiac oxidative stress and fibrosis: decreased plasma and cardiac MDA, increased cardiac content of the antioxidant enzymes GSH and SOD activity, decreased levels of pro-inflammatory cytokines (TNF-α, IL-1β and IL-6). Substantial inhibition of the TGF-β1/Smad2 and 3 signaling pathway after 6 weeks treatment (*p* < 0.05).	In vivo study 44 male Sprague Dawley rats	Increases insulin sensitivity, reduces diabetic neuropathy	[126]
Gliclazide 15 mg/kg	Alpha lipoic acid (ALA) 60 mg/kg	Supplementation with ALA reduced apoptosis by 6% and restored cell viability to a level comparable to the healthy control (HC) group, while gliclazide further reduced cell viability and significantly increased apoptosis by 29% compared to the HC group (*p* > 0.05). Supplementation with ALA preserved hepatic integrity against inflammation and apoptosis (*p* < 0.001).	Preclinic In vivo study	Suppression of inflammation and apoptosis by activating the antioxidant pathway in the diabetic liver	[127]
Gliclazide 2.5/5.6/11.2 mg/kg	Resveratrol 50/100/200 mg/kg	This combined therapy significantly enhanced the mean percent blood glucose reduction at the 4th h in both normal (44.44% ± 1.09%) and diabetic rats (52.67% ± 0.32%) without biphasic hypoglycemia. In both single and multiple dose combinations, co-administration of gliclazide with resveratrol resulted in significant increases to pharmacokinetic parameters, such as AUC (Area under serum concentration/time), C_max_ (Peak serum concentration), and t_1/2_ (Terminal half life), while reducing Clearance (CL), Vd (Volume of distribution), and Vd_ss_ compared to gliclazide alone.	In vivo study albino rabbits and adult male albino Wistar rats	substantial changes in blood glucose reduction in normal rats, diabetic rats, and normal rabbits	[128]
Glibenclamide 600 μg/kg	Honey 1.0 g/kg/day	No significant differences in the activities of SOD, GSH in pancreas of normal and STZ-treated rats. Only Glibenclamide did not increase CAT activity in diabetic rats, but glibenclamide in combination with honey showed significantly (*p* ≤ 0.016) increased CAT and (*p* ≤ 0.016) reduced GPx activity.	In vivo study rats	Blood glucose was reduced reduced activity of catalase increased activities of superoxide dismutase and glutathione peroxidase	[129]
Glibenclamide 5 mg/day	Sesame oil 35 g/day	Antihyperglicemic effect: combination therapy showed 36% reduction of glucose (*p* < 0.001 vs. before treatment, *p* < 0.01 vs. sesame oil monotherapy, *p* < 0.05 vs. glibenclamide monotherapy) and 3% reduction of HbA1c (*p* < 0.001 vs. before treatment, *p* < 0.01 vs. sesame oil monotherapy, *p* < 0.05 vs. glibenclamide monotherapy)	In vivo study 60 diabetic patients, underwent combination therapy	Decreases lipid peroxidation, protects pancreatic β-cells	[130]
Glimepiride 0.1 mg/kg	Naringin 100 mg/kg	Combination therapy significantly restores the creatinine levels and urine volumes, SGOT (serum glutamate oxaloacetate transaminase), SGPT (erum glutamate pyruvatetransaminase), and ALP when compared to a single dose of administration (*p* < 0.001). The following biochemical parameters TC (total cholesterol), LDL, TG and HDL, as well as CAT, GSH, SOD endogenous antioxidant enzymes, were significantly returned to normal levels.	In vivo study Wistar rats	Lipid normalization Lower blood sugar Increased antioxidant enzymes	[131]
Glimepiride 1 mg/kg	Curcumin 80 mg/kg	In diabetic rats: increased all the pharmacokinetic parameters including C_max_ (by 1.37 times), AUC (by 1.16 times), t_1/2_ (by 1.68 times) and MRT (Average mean residence time—by 1.29 times), decreased the CL (by 0.68 times), Vd (by 0.61 times) markedly as compared to control group.	In vivo study Male Albino rats of Wistar	Decreases glucose levels and systemic inflammation	[132]
Glipizide 15 mg/day	Aralia root bark extract (ARBE) 2.7 g/day	Compared with the glipizide group, the combination group had significant decreases levels of HbA1c (*p* = 0.06), TC (*r* = 0.32; *p* = 0.006) and LDL-C (*r* = 0.34; *p* = 0.005).	148 patients treated for 8 weeks	decrease in HbA1c and LDL-C levels	[133]
Other SU combinations					
Glibenclamide 10 mg/day	Metformin (Glucovance, producer Merck Serono) 1000 mg/day	Serum concentration of AGE (Advanced Glycation Products), AOPP (Advanced Oxidation Protein Product), MDA and Ox-LDL (Oxidized Low-Density Lipoprotei) decreased significantly (*p* < 0.001) compared to baseline. Enzymatic activities of CAT, FRAP, GPX, and SOD increased significantly (*p* < 0.001). Increased HbA1c compared to metformin group (7.5 ± 1.7 vs. 6.8 ± 0.9, *p* = 0.010)	In vivo study 95 patients	Reduction of OS markers (MDA, LDL), increase in antioxidant enzymes SOD, CAT, and GPx.	[134]
Glipizide 2.5 mg/kg	Metformin (Metaglip, producer Bristol-Myers Squibb) 60 mg/kg	Compared to the untreated DM group, FBG and HbA1c were significantly reduced in the DM groups (*p* < 0.01) treated with metformin (159.7 mg/dL and 6.7%), glipizide (184.3 mg/dL and 7.3%) and dual therapy (118 mg/dL and 5.2%). Dual therapy decreased LPO, NO and urea levels but increased TAC in diabetic rats more than glipizide.	In vivo study 30 Sprague Dawley rats male	Lipid peroxidation and NO levels decreased, while TPAC increased.	[83]
Gliclazide 80 mg	Metformin (Claformin, producer Orchid Pharma) 500 mg	Monotherapy with metformin showed significantly lower levels of FBG [7.61 (6.70–8.89) mmol/L vs. 9.00 (7.30–10.68) mmol/L; *p* = 0.022] and HBA1c [7.00 (6.40–7.65)% vs. 8.20 (7.20–9.75)%; *p* < 0.001] compared with dual therapy.	In vivo study 80 patients, only 40 underwent combination therapy	Decrease in HbA1c, increase in TPAC	[135]
Glimepiride 0.2 mg/kg	Pioglitazone (thiazolidinedione) 1 mg/kg	This dual therapy did not protect from nuclear damage and sperm abnormalities in NA-STZ diabetes compared to the combination of metformin and pioglitazone.	In vivo study Wistar rats male	Reduction in LPO Increase in enzyme production (CAT and SOD) Reduction of OS and hyperglycemia	[136]
Glimepiride 1 mg	Dapaglifozina 10 mg + clorhidrat de metformin 1000 mg	12 week: The reduction in HbA1c from baseline was statistically significant with the triple therapy as compared to dual therapy (glimepiride and metformin) (mean ±SD: −1.37% ± 0.93% vs. −1.01% ± 0.79%; *p <* 0.0001) 28 week: the reduction of HbA1c was comparable between the two types of therapies (−2.08 ± 1.06% vs. −1.80% ± 1.07%, *p =* 0.4943)	In vivo study randomized phase III clinical trial	Reduction in HbA1c Decreases of ROS and NOX_4_	[137]
Gliclazide 10 mg/kg	Atorvastatin 10 mg/kg	Combination treatment enhanced sperm morphology and improved testicular structure (*p* < 0.05) while did not affect sperm count (*p* > 0.05).	In vivo study Sprague-Dawley rats male	Increased antidiabetic properties Independent antioxidant properties Gliclazide decreases NADPH oxidase Atorvastatin activates CAT and induces NFR2/GPX_4_	[138]

Triple therapy with glimepiride, dapagliflozin, and metformin hydrochloride is the latest study developed in 2025 for patients with T2DM, a phase III randomized clinical trial conducted by Rakesh Sahay DM et al. Eligible patients, selected from the Indian region, were tested and monitored for 30 weeks. Half of them received dual therapy (glimepiride and metformin) and the other half received triple therapy (glimepiride, metformin, dapaglifozin). The objective of the study was to reduce HbA1c, FBG (fasting blood glucose), and PPBG (postprandial blood glucose), which was achieved by the triple combination and supported by reports in the literature where a decrease in HbA1c is associated with an increase in adherence to oral antidiabetic medication. This paper suggests that one of the factors leading to suboptimal blood glucose control is non-adherence to treatment and polypharmacy, which is why a triple combination in a single pill taken once a day offers diabetic patients comfort, efficacy, and increased adherence [138]. Consequently, promising results for associated therapies can be found in the literature, contributing to the optimization of management strategies for patients suffering from T2DM associated with OS [139,140,141,142,143].

The observed synergy between SU and antioxidants is crucial, particularly in how these combinations interact at the metabolic pathway level, resulting in significant therapeutic benefits for managing T2DM [144]. Therefore, the molecular and cellular mechanisms that underlie the synergy between SU and natural antioxidants involve the PI3K/Akt/GLUT4 pathway, the AMPK (AMP-activated protein kinase) pathway, and a reduction in NF-kB activation [145]. SU stimulates insulin secretion by blocking K^+^-ATP channels on the β-cell membrane. However, long-term use of SU is associated with increased oxidative stress and cell apoptosis [146]. Antioxidants like quercetin, ALA, curcumin, and resveratrol counteract the damaging effects of ROS by neutralizing them [123,127,129]. This process helps protect pancreatic β-cells and extend their functional viability. In terms of cellular signaling, antioxidants play a crucial role in activating the PI3K/Akt pathway, which restores insulin sensitivity and promotes the translocation of the GLUT4 transporter in muscle and fat cells. Additionally, by modulating the activation of NF-kB and the expression of pro-inflammatory cytokines such as IL-6 and TNF-α, antioxidants help reduce chronic inflammation associated with insulin resistance. Furthermore, the activation of the AMPK pathway by antioxidants enhances glucose uptake and inhibits gluconeogenesis in the liver [147].

In conclusion, the combination of SU with antioxidants provides synergistic benefits, including protection of pancreatic β-cells from oxidative damage, enhancement of insulin signaling pathways, reduction of inflammation and insulin resistance, mitigation of toxicity related to SU treatment, and a decrease in adverse effects. This approach holds great promise for improving glycemic control.

## 3. Conclusions and Perspectives

Understanding the pathogenesis of Type 2 Diabetes Mellitus and its complications is crucial, particularly regarding the role of oxidative stress in damaging pancreatic β-cells and contributing to insulin resistance. It is important to explore the molecular mechanisms involved, especially the significance of mitochondria in generating oxidative stress in diabetes. Additionally, identifying biomarkers of oxidative stress is essential for preventing the progression of this disease. Given the multifactorial nature of metabolic syndrome, there is a pressing need for a multi-targeted approach to treatment. Clinical translational research is a dynamic field that connects laboratory findings with patient care. This area of research offers significant benefits for patients with Type 2 Diabetes Mellitus and oxidative stress, including new treatment options, opportunities for earlier diagnosis, and a reduction in complications. This highlights the importance of advancing research and developing therapies that focus on personalizing treatment based on the molecular profiles of diabetic patients. However, translational research faces several challenges, including the high costs of clinical trials, the lengthy timeline from discovery to the application of therapies, stringent regulations, and the frequent occurrence of failures during the clinical phase. Particularly challenging are the issues associated with the development of Traditional Chinese Medicine (TCM) preparations. The variability of TCM medicinal plants can lead to inconsistencies in active compounds, which in turn affects both the efficacy and safety of the products. Additionally, the absence of unique chemical markers complicates quality control for TCM preparations, unlike modern drugs that have clearly defined active ingredients. TCM formulations can offer both benefits and risks, making it difficult to achieve consistent results. Therefore, TCM preparations require well-established quality control measures, including tests for purity, contamination, and the identification of active substances. The standardization of production processes is crucial; without strict protocols, the chemical composition can vary significantly. TCM typically employs methods such as decoctions and infusions, which can result in synergistic formulas that are challenging to dose, test, and validate effectively. On the other hand, it is essential to pay special attention to the interaction between Traditional Chinese Medicine (TCM) and Western (allopathic) medicine. This focus is important for several reasons, including therapeutic efficacy, patient safety (particularly in avoiding cumulative toxicity, drug interactions, and uncontrolled adverse effects) and the development of a holistic and personalized approach to chronic diseases such as Type 2 Diabetes Mellitus (T2DM). These limitations highlight the need for a synergistic approach, as discussed in this review, which explores the combination of synthetic compounds with proven antidiabetic effects, supported by extensive studies that have progressed through all clinical phases, alongside a variety of natural antioxidant compounds.

This study place on the effective management of Type 2 Diabetes Mellitus associated with oxidative stress using one of the most recommended and prescribed classes of drugs, namely sulfonylureas. Since their initial discovery and through their synthetic development and testing over the years, sulfonylureas have been tested both individually and in combination with other natural compounds, such as antioxidants, other antidiabetics, or lipid-lowering agents, demonstrating substantial effects on the glycemic profile, oxidative stress, and lipid profile.

Therefore, the purpose and relevance of explaining the evidence extracted from the literature are highlighted by the centralization of new synergistic therapies to improve diagnosis, prevent complications, and identify future synthetic pathways to achieve promising therapeutic targets, treat DM and associated dysfunctions, such as OS. The reduced risk of complications and the demonstrated synergistic potential identified in preclinical and clinical research through in vivo studies are just a few advantages that underscore the importance of this research. These studies have shown that various therapies can enhance the activity of antioxidant enzymes, such as superoxide dismutase, glutathione peroxidase, and catalase. Additionally, they can lower levels of inflammatory markers, reduce lipid peroxidation, and decrease DNA oxidation.

Moreover, investigating these combination therapies through in vitro, in silico and in vivo studies would greatly enhance validation and provide a complementary approach for future research. The unexplored antioxidant potential of certain sulfonylureas, like tolazamide and acetohexamide, also presents interesting opportunities for further scientific investigation. Future research should investigate how antioxidants influence gene expression related to the PI3K/Akt, AMPK, and NF-kB pathways, as well as their effects on mitochondrial stress and redox pathways linked to pancreatic β-cell apoptosis in the presence of sulfonylureas. Additionally, it is essential to enhance pharmacodynamic and pharmacokinetic studies of these combinations to optimize dosages and prevent negative interactions. Other studies could focus on controlled human trials that measure HbA1c levels, systemic oxidative stress, and microvascular complications. Given that Type 2 Diabetes Mellitus (T2DM) may be connected with cognitive decline and neuropathy, valuable research could also explore the beneficial effects of combining sulfonylureas and antioxidants on the insulin-brain axis, assessing their neuroprotective properties. These perspectives could significantly contribute to advancements in both scientific and medical fields.

## Data Availability

No new data were created.

## Abbreviations

The following abbreviations are used in this manuscript:

DM	Diabet Mellitus
T1DM	Type one Diabet Mellitus
T2DM	Type two Diabet Mellitus
OS	Oxidative Stress
ROS	Reactive Oxygen Species
RNS	Reactive Azote Species
LP	Lipid peroxidation
PG	Protein glycosylation
TZD	Thiazolidinedione
DPP-4	Dipeptidyl peptidase-4 inhibitor
SGLT-2	Sodium-Glucose Transport Protein 2 Inhibitors
GLP-1	Glucagon-like peptide-1
AGEs	Advanced glycation end products
PKC	Protein kinase C
MDA	Malondialdehyde
GSH/GSSG	Glutathione peroxidase
SOD	Superoxide dismutase
CAT	Catalase
NADPH	Nicotinamide adenine dinucleotide phosphate oxidase
SU	Sulfonylurea
HgA1c	Glycosylated hemoglobin
SUR	Sulfonylurea receptor
OGTT	Oral glucose tolerance test
ITT	Insulin tolerance test
DPPH	2,2-diphenyl-1-picrylhydrazyl
FRAP	Ferric reducing antioxidant power
ORAC	Oxygen radical absorbance capacity
ABTS	2,2′-azinobis(3-ethylbenzothiazoline-6-sulfonic acid)
TPAC	Total plasma antioxidant capacity
LDL	Low-density lipoprotein
HDL	High-density lipoprotein
NO	Nitric oxide
FBG	Fasting blood glucose
PPG	Postprandial blood glucose
GPx	Glutathione peroxidase
ATP	Adenosine Triphosphate
IRS-1	Insulin Receptor Substrate-1
ER	Endoplasmic Reticulum
NF-kB	kappa B Nuclear Factor
UPR	Unfolded Protein Response
